# Pulmonary granulomas confirmed in Blau syndrome using TBLC specimens: Case report

**DOI:** 10.3389/fmed.2024.1380236

**Published:** 2024-06-12

**Authors:** Yasuo Shimizu, Yoshitomo Kushima, Ayae Tanaka, Akihiro Takemasa, Kazuyuki Ishida, Seiji Niho

**Affiliations:** ^1^Department of Pulmonary Medicine and Clinical Immunology, Dokkyo Medical University, Mibu, Tochigi, Japan; ^2^Respiratory Endoscopy Center, Dokkyo Medical University Hospital, Mibu, Japan; ^3^Center of Regenerative Medicine, Dokkyo Medical University Hospital, Mibu, Japan; ^4^Department of Rheumatology, Dokkyo Medical University, Mibu, Japan; ^5^Department of Diagnostic Pathology, Dokkyo Medical University, Tochigi, Japan

**Keywords:** *NOD2*, cryobiopsy, lung, sarcoidosis, granulomas, Blau syndrome, bronchoscopy, interstitial lung disease

## Abstract

Blau syndrome (BS), is an autoinflammatory granulomatosis disease characterized by a distinct triad of skin, joint, and eye disorders similar to those of sarcoidosis, but the lung involvement frequently observed in sarcoidosis are rare. Granulomas from patients with BS displayed a distinct morphology indicating an exuberant chronic inflammatory response. Patients with BS may have granulomatous lung lesions, which require early diagnosis. To determine whether therapeutic intervention is needed for lung lesions, examining transbronchial lung cryobiopsy specimens and accumulating cases of BS with lung involvement could be contributed to improving BS management in the future.

## Introduction

Juvenile sarcoidosis, known as Blau syndrome (BS), is an autoinflammatory granulomatosis disease characterized by a distinct triad of skin, joint, and eye disorders (1). Nucleotide-binding oligomerization domain 2 (*NOD2)* has been identified as the gene responsible for this disease, and the autosomal dominant or sporadic gene mutations induce autoactivation of nuclear factor kappa B (2).

The symptoms of BS are similar to those of sarcoidosis, but the lymphadenopathy and lung involvement frequently observed in sarcoidosis are rare, and the first case of interstitial lung disease in BS was reported in 2007 (3). In the present case, the patient had early-onset BS at the age of 2 years, and the M513T mutation in the *NOD2* was identified and previously reported (2). Transbronchial lung cryobiopsy (TBLC) was performed on the tiny but diffuse granular shadows in the lungs, and pathological examination of the specimens confirmed the granulomas. There have been very few reports of lung lesions in BS, and to date, no report has pathologically proven granulomas in the lung and adult-onset lung granulomas in BS.

## Case presentation

**FIGURE 1:**
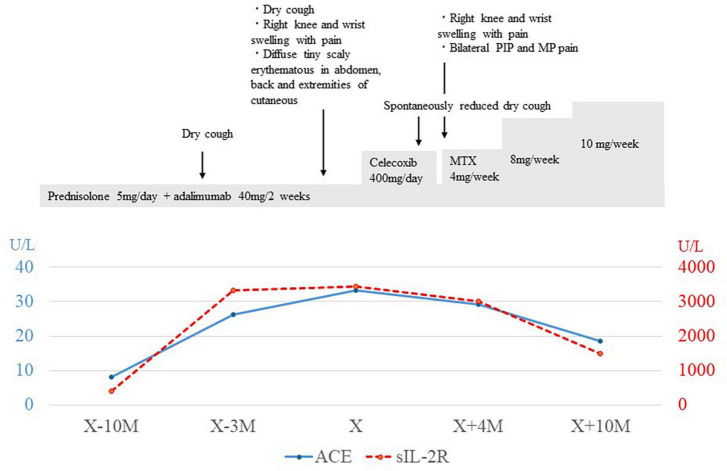
Clinical and treatment course. X indicated the day of bronchoscopic examination was performed, and X-10M indicated the day of 10 month (M) before the day of bronchoscopic examination was performed. Changes in serum levels of angiotensin converting enzyme (ACE) and soluble interleukin-2 receptor (sIL-2R) were in blue line and red-dot line, each. PIP, proximal interphalangeal; MP, metacarpophalangeal; MTX, Methotrexate.

Herein, a 22-years-old man was referred to our department for the examination of extensive bilateral lung granular shadows. On admission to receive TBLC, the vital signs of the patient were body temperature of 36.3°C, regular pulse of 75 bpm, SpO_2_ of 99% in room air, and auscultation for the heart and lungs was normal. He had been having dry cough for 3 months before bronchoscopic examination (the day X-3M) (Figure 1), and painful swelling of the right knee and right wrist, and diffuse tiny scaly erythematous in abdomen, back and extremities of cutaneous with mild pigmentation, were accompanied with emergence of cough.

**FIGURE 2:**
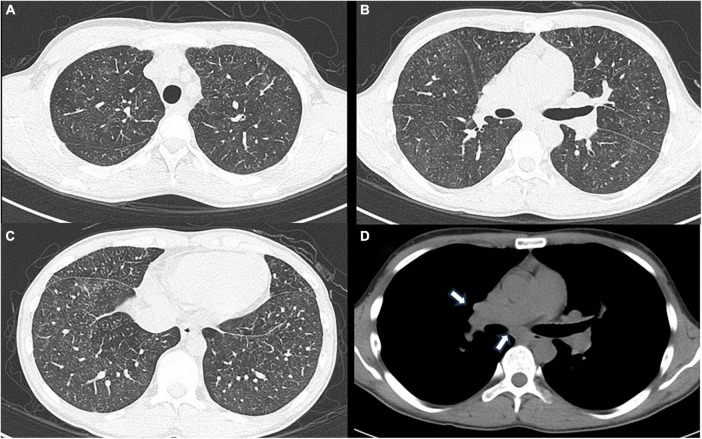
**(A)** Chest computed tomography using the lung window setting shows diffuse ground-glass shadows presumed to be fine nodules in the bilateral upper lobes, **(B)** middle and lingular lobes, **(C)** and bilateral lower lobes. **(D)** Chest computed tomography using the mediastinum window setting shows minor swelling of mediastinal lymph nodes, indicated by white arrows.

Laboratory examination revealed a normal range of leukocytes (4800 cells/μL) and fraction of neutrophils (56%), eosinophils (2.9%), basophils (1.0%), monocytes (16.9%), and lymphocytes (23.2%), but elevated angiotensin-converting enzyme (33.4 U/L), soluble interleukin 2 receptor (3440 U/ml), and matrix metalloproteinase-3 (233 ng/ml). The electrocardiogram showed incomplete right bundle branch block, spirometry showed a normal range of forced vital capacity (83.7%), forced expiratory volume in 1 second (83.1%), and DLCO (84.6%). Chest X-rays and CT scans showed diffuse fine granular shadows extending into the bilateral lungs (Figures 2A–C) and minor swelling of the mediastinal lymph nodes (Figure 2D).

The patient was treated with prednisolone (5–10 mg/day) and human IgG1 monoclonal antibody specific for tumor necrosis factor-α (TNF-α, adalimumab) at a dose of 40 mg once every two weeks, which was introduced 2 years ago to treat arthritis and reduce the adverse effects of steroids. Differential diagnosis for granular shadows in the bilateral lungs suggested fungal and acid-fast bacillus infections, drug-induced granulomatosis, lymphoma, vasculitis, and granulomas due to BS.

**FIGURE 3:**
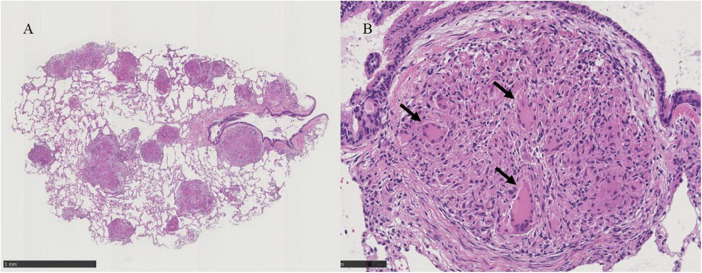
**(A)** Histological examinations of the lung tissue obtained by transbronchial lung cryobiopsy show non-caseating epithelioid granulomas scattered around the alveolar septum, bronchi, vessels and large complex of granulomas without inter granulomatous sclerosis at X30 magnification, black scale bar = 1mm. **(B)** Prominent epithelioid cells and multinucleated giant cells around bronchioles are indicated by black arrows in the panel at X200 magnification, black scale bar = 100 μm.

Bronchoscopy was performed on the lesions of the lung field and mediastinal lymph nodes on day X (Figure 1). The lumen of the main bronchus showed reticulated dilation of the capillaries, appearing like sarcoidosis. Bronchoalveolar lavage fluid (BALF) was collected from the left B^5^a, and 101/150 mL of lavage was recovered. The cell fraction of BALF was predominantly lymphocytes (87%), and the CD4/8 ratio was not elevated (0.97). Transbronchial needle biopsy (TBNA) was performed on longitudinal lymph nodes #7 and #2R, where access was feasible. TBLC was performed on right B^8^a, right B^4^a, and right B^3^a. In a previous report, granulomas from patients with BS displayed a distinct morphology characterized by large polycyclic granulomas with dense lymphocytic coronas, forming a large granulomatous complex without inter granulomatous sclerosis, indicating an exuberant chronic inflammatory response (4). In the present case, specimens obtained by TBLC were sufficiently large to observe complex of granulomas and inter granulomas, and those specimens showed non-caseating epithelioid granulomas scattered around the alveolar septum, bronchi, and vessels forming a large granulomatous complex without inter granulomatous sclerosis at low magnification (Figure 3A). At high magnification, prominent epithelioid cells and multinucleated giant cells around bronchioles were observed, similar to previous reports on polycyclic granuloma observed in lymph nodes or skin biopsy specimens from BS patients (4), but in the present case, they were not seen in granulomas with dense lymphocytic coronas (Figure 3B). TBNA specimens contained epithelioid cells. The cultures of cells obtained from saline for washing needle of TBNA and for probe of TBLC, BALF and an aspirated sputum sample through bronchoscopy did not reveal bacterial or fungus infections and mycobacterial infection by Ziehl-Neelsen stain and culture using mycobacterium growth indicator tube (MGIT) system. Based on clinical, hematological, and pathological findings, the lung lesions were diagnosed as pulmonary granulomas caused by BS. While an additional dose of prednisolone was considered for the therapy of cough and lung lesions, taking into account the lack of SpO2 decrease, the patient’s young age, the increased risk of adverse effects from additional prednisolone, the absence of infection and also the possibility of spontaneous resolution of granulomatous lesions, the patient was followed up for 2 months without escalating dose of prednisolone or additional immunosuppressant. Celecoxib, a cox-2 inhibitor, was additionally administered for arthralgia of the knees and wrists, and arthralgia was alleviated. Cough gradually decreased spontaneously subsequent two months (X+2M), and chest X-ray also showed a slight improving. However, after four months form bronchoscopic examination (X+4M), right knee and right wrist, proximal interphalangeal (PIP) joint pain and metacarpophalangeal (MP) joint pain were aggravated again. Since chief complaint of the patient was joint pain and prednisolone and methotrexate (MTX) and/or azathioprine were recommended for pulmonary sarcoidosis (5), MTX 4mg/2weeks was started for the arthralgia. The dose was subsequently increased, and pain was under control. No adverse effects due to MTX have appeared. Despite worsening arthralgia on the day X+4, cough and the lung lesions have remained without recurrence, and those were no subsequent deterioration. Changes in serum angiotensin converting enzyme (ACE) and soluble interleukin-2 receptor (sIL-2R) levels appeared to reflect well the manifestation of cough and lung lesions and their subsequent resolution (Figure 1).

## Discussion

Blau syndrome is characterized by dermatitis, arthritis, and uveitis related to *NOD2* mutation, but in recent years, granulomatous lesions other than the triad of BS have also been considered as a phenotype of BS (3). The background of the manifestation of lung lesions in this patient was speculated from three aspects. The first was whether the frequency of pulmonary lesions differed depending on the type of NOD2 mutations, the second was whether there was a trigger for enhancing *NOD2* pathway activation, and the third was whether there were triggers for pulmonary lesion formation observed in adult sarcoidosis. First, BS patients who have been reported to having pulmonary lesions with *NOD2* mutation were a 23-years-old male with amino acid substitution Glu498Gly (not R334Q, R334W and L489F) (6), a six-teen-years-old male with R334 Q (3), a two-years-old and 7 months boy with pulmonary hemorrhage due to bronchial granuloma with the R334Q, a two-years-old boy with R334W and a nineteen-year-old male of this presenting with M513T. R334W is most common in BS and R334Q is also frequent but M513T is less frequent in BS (7). The activation levels of NF-κb by NOD2 mutations are similar among R334Q, R334W and M513T (7). In terms of these, it is not possible to determine the mutation that predispose to pulmonary lesions in BS. Second, NOD2 mutation leads to autoactivation of NF-κb, but external factors might further amplify the activation of the pathway from *NOD2* to NF-κb. *NOD2* is activated by muramyl-dipeptide MurNAc-L-Ala-D-isoGln (MDP), a conserved proteoglycan (PGN) found in almost all bacteria and also mycobacterium tuberculosis activates via MDP. Single strand RNA virus including influenza A or RS virus induce NOD2 expression (8). In the presenting patient, there was no tuberculosis or influenza infection at the emergence of the pulmonary lesions, but RS virus infection was not examined, so the involvement of RS virus infection could not be ruled out as a trigger for developing lung lesions in present case. BCG vaccination also activates NOD2 (7), but there was no BCG vaccination in this patient. There is a report that propionibacterium acnes (*P. acnes*) had been detected on the skin specimen of a patient with BS (9), but whether *P. acnes* was involved in pulmonary lesion was unknown because staining using anti-propionibacterium acnes monoclonal antibody (PAB) was not performed on specimens of present patient. Third, Mycobacterium tuberculosis, cutibacterium acnes, metals, carbon, silicon inorganic antigens could be antigen for sarcoidosis (10), but there was no history of inhalation of these antigens in present case. Examination with polarized light to rule out foreign body was not done on specimens.

In an *ex vivo* study using pluripotent stem cells from BS patients with the *NOD2* R334W mutation, abnormal cytokine expressions in monocytes were induced by interferon-γ stimulation, and those expressions were inhibited by anti-TNF-α antibody or pan Janus kinase inhibitor, tofacitinib (11, 12). In the present case, despite the therapy with prednisolone and adalimumab, lung lesions appeared accompanied with worsening arthralgia and skin rash, but at the time of recurrence of worsening arthralgia, cough and lung lesion did not appear. MTX administration was started at the time of recurrence of arthralgia and MTX has been continued since then, and there has been no recurrence of lung lesions. It was unclear whether MTX contributed to the suppression of recurrence of pulmonary symptoms and lesions. In two previous case reports of pulmonary involvement in adolescent (16-years-old) and adult (23-years-old) BS, one case report of adult BS recommended the use of prednisolone and immunosuppressive agents (6). In another 16-years-old BS case, disease control was achieved with prednisolone 10 mg/day combined with anti-TNF-α (infiliximab) 10 mg/kg every 8 weeks (3). In that case report, biopsy of the lung lesion was not performed, but a biopsy was performed on the posterior auricular lymph node. The pathological findings demonstrated discrete non-necrotizing granulomas with a multinucleated cell, but large granulomatous complexes without inter-granulomatous sclerosis, which could be found in present case by TBLC were difficult to determine from the literature (3). In presented case, when pulmonary symptoms and granulomas appear again, it may be useful to monitor the changes in ACE and sIL-2R levels and consider the use of immunosuppressive agents if the pulmonary lesions worsen.

Unlike sarcoidosis, the spontaneous remission of BS is rare, and the syndrome eventually leads to sequelae such as blindness and joint contractures. Thus, early diagnosis are considered necessary, even in the lungs. The diagnostic rate was reported to be 37–90% for transbronchial lung biopsy (TBLB) and 80–92% for TBLC in sarcoidosis, a major disease presenting with granulomatous lesions of the lungs (13, 14). The similarities and differences between pulmonary granulomatous lesions in BS and sarcoidosis remain unresolved.

Accordingly, accumulating knowledge from pathological examination on TBLC specimens would be useful in determining the timing of therapeutic intervention for lung lesion and that can contribute to improving management for BS in the future.

## Data availability statement

The original contributions presented in the study are included in the article/supplementary material, further inquiries can be directed to the corresponding author.

## Ethics statement

Written informed consent was obtained from the patient for the publication of any potentially identifiable images or data included in this article.

## Author contributions

YS: Writing−review and editing, Writing−original draft, Visualization, Project administration, Investigation, Formal analysis, Data curation, Conceptualization. YK: Writing−review and editing, Writing−original draft, Investigation, Data curation. AyT: Writing−review and editing, Writing−original draft, Data curation. AkT: Writing−review and editing, Writing−original draft, Methodology. KI: Investigation, Writing−review and editing, Writing−original draft, Visualization, Validation. SN: Writing−review and editing, Writing−original draft, Supervision.
